# Supplementary material for: Causal Relationship between Aging and Anorexia Nervosa: A White-Matter-Microstructure-Mediated Mendelian Randomization Analysis
Source: Biomedicines. 2024 Aug 16;12(8):1874. doi: 10.3390/biomedicines12081874 (PMC11351342; doi:10.3390/biomedicines12081874)
Supplement: Supplementary file 1 [file biomedicines-12-01874-s001.zip › Supplementary Figure/Figure S4/Singe SNP plot of MR analysis for TL to ubm-b-2078.pdf]

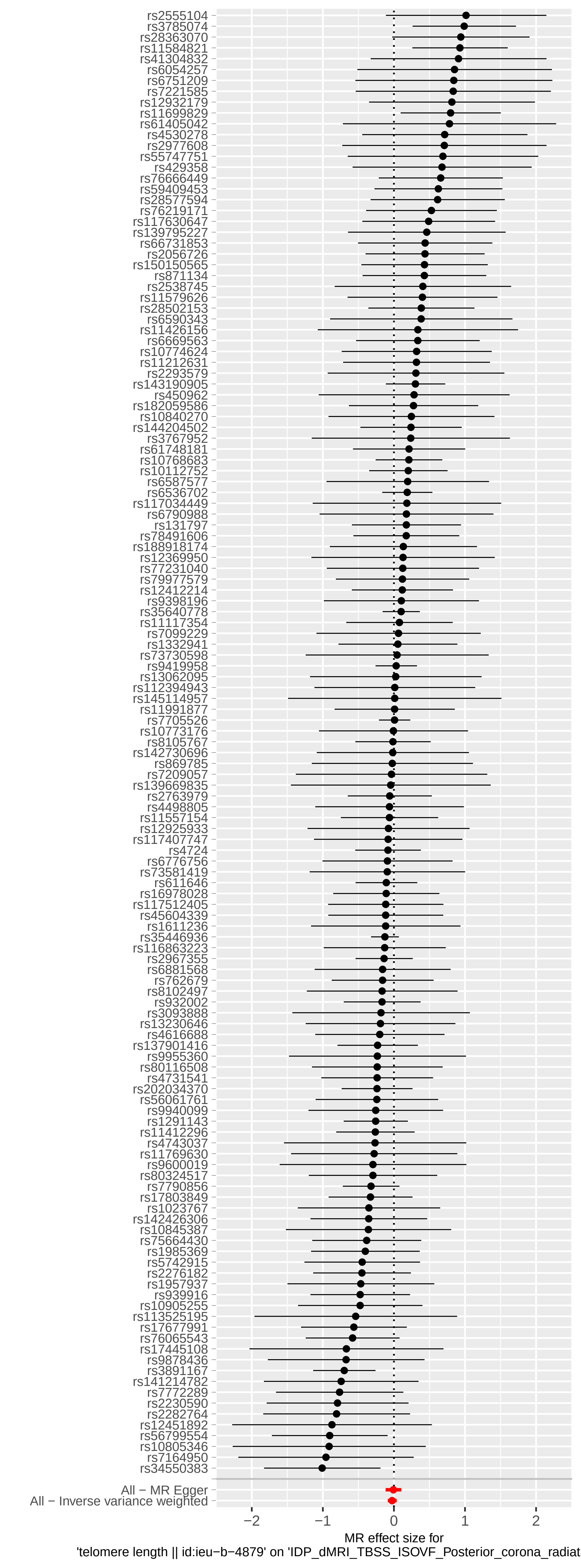
rs2555104
rs3785074
rs28363070
rs11584821
rs41304832
rs6054257
rs6751209
rs7221585
rs12932179
rs11699829
rs61405042
rs4530278
rs2977608
rs55747751
rs429358
rs76666449
rs59409453
rs28577594
rs76219171
rs117630647
rs139795227
rs66731853
rs2056726
rs150150565
rs871134
rs2538745
rs11579626
rs28502153
rs6590343
rs11426156
rs6669563
rs10774624
rs11212631
rs2293579
rs143190905
rs450962
rs182059586
rs10840270
rs144204502
rs3767952
rs61748181
rs10768683
rs10112752
rs6587577
rs6536702
rs117034449
rs6790988
rs131797
rs78491606
rs188918174
rs12369950
rs77231040
rs79977579
rs12412214
rs9398196
rs35640778
rs11117354
rs7099229
rs1332941
rs73730598
rs9419958
rs13062095
rs112394943
rs145114957
rs11991877
rs7705526
rs10773176
rs8105767
rs142730696
rs869785
rs7209057
rs139669835
rs2763979
rs4498805
rs11557154
rs12925933
rs117407747
rs4724
rs6776756
rs73581419
rs611646
rs16978028
rs117512405
rs45604339
rs1611236
rs35446936
rs116863223
rs2967355
rs6881568
rs762679
rs8102497
rs932002
rs3093888
rs13230646
rs4616688
rs137901416
rs9955360
rs80116508
rs4731541
rs202034370
rs56061761
rs9940099
rs1291143
rs11412296
rs4743037
rs11769630
rs9600019
rs80324517
rs7790856
rs17803849
rs1023767
rs142426306
rs10845387
rs75664430
rs1985369
rs5742915
rs2276182
rs1957937
rs939916
rs10905255
rs113525195
rs17677991
rs76065543
rs17445108
rs9878436
rs3891167
rs141214782
rs7772289
rs2230590
rs2282764
rs12451892
rs56799554
rs10805346
rs7164950
rs34550383
All − MR Egger
All − Inverse variance weighted
−2
−1
0
1
2
MR effect size for
'telomere length || id:ieu−b−4879' on 'IDP_dMRI_TBSS_ISOVF_Posterior_corona_radiat
